# Obesity and Its Potential Effects on Antidepressant Treatment Outcomes in Patients with Depressive Disorders: A Literature Review

**DOI:** 10.3390/ijms17010080

**Published:** 2016-01-12

**Authors:** Young Sup Woo, Hye-Jin Seo, Roger S. McIntyre, Won-Myong Bahk

**Affiliations:** 1 Department of Psychiatry, College of Medicine, the Catholic University of Korea, Seoul 07345, Korea; youngwool@catholic.ac.kr (Y.S.W.); carpe-diem80@daum.net (H.-J.S.); 2 Mood Disorders Psychopharmacology Unit, University Health Network, Toronto, ON M5T 2S8, Canada; Roger.McIntyre@uhn.ca; 3 Department of Psychiatry, University of Toronto, Toronto, ON M5T 2S8, Canada

**Keywords:** depressive disorder, obesity, antidepressant, response, treatment outcome

## Abstract

Accumulating evidence regarding clinical, neurobiological, genetic, and environmental factors suggests a bidirectional link between obesity and depressive disorders. Although a few studies have investigated the link between obesity/excess body weight and the response to antidepressants in depressive disorders, the effect of weight on treatment response remains poorly understood. In this review, we summarized recent data regarding the relationship between the response to antidepressants and obesity/excess body weight in clinical studies of patients with depressive disorders. Although several studies indicated an association between obesity/excess body weight and poor antidepressant responses, it is difficult to draw definitive conclusions due to the variability of subject composition and methodological differences among studies. Especially, differences in sex, age and menopausal status, depressive symptom subtypes, and antidepressants administered may have caused inconsistencies in the results among studies. The relationship between obesity/excess body weight and antidepressant responses should be investigated further in high-powered studies addressing the differential effects on subject characteristics and treatment. Moreover, future research should focus on the roles of mediating factors, such as inflammatory markers and neurocognitive performance, which may alter the antidepressant treatment outcome in patients with comorbid obesity and depressive disorder.

## 1. Introduction

Despite the introduction of a number of antidepressant drugs and their prolonged clinical use, many patients with depression show an inadequate response to standard antidepressant therapy [1,2,3,4]. Furthermore, the high levels of interindividual variability in the responses to individual antidepressants render treatment outcomes uncertain. As unresolved depression is associated with high morbidity and has a negative impact on health care utilization and costs due to the extensive use of medical services, it is important to identify the risk factors of poor treatment outcome. Moreover, predictors of responses to specific antidepressants or within a specific population are also needed to allow appropriate treatment planning in individual cases [5].

However, although a number of factors that potentially increase the likelihood of an inadequate response and contribute to high interindividual differences in the response to antidepressants have been discussed, there are no reliable predictors of the clinical response to antidepressants [6,7,8]. Among the possible predictors of treatment response in depressive disorder, obesity may contribute to a poor treatment response [9]. Several studies suggest that a higher relative body weight or obesity may predict a poor outcome to antidepressant treatment [10,11,12,13,14]. However, these studies differed in subject demographic and clinical characteristics, study design and duration, antidepressants administered, and definition and measures of obesity/excessive body weight. In addition, a few studies found no association between obesity/body weight and treatment response [15,16,17]. These differences and discrepancies preclude reaching definitive conclusions based on these previous studies.

If obesity or excess body weight is shown to be a reliable predictor of treatment outcome in patients with depressive disorder, body weight and body mass index (BMI) could be useful and plausible measures, because they are easily obtained and are likely to influence drug responses through pharmacokinetic and pharmacodynamics mechanisms [18]. Although Mansur *et al.* [19] reviewed the literature extensively regarding the relationship between obesity and mood disorders, they focused mainly on the potential role of obesity in the pathophysiology of mood disorders, and the association between obesity/excess body weight and treatment outcomes remains unclear.

The aims of this literature review were to discuss the relationship between obesity/excess body weight and the outcome of antidepressant therapy in patients with depressive disorders, and to investigate the possibility of obesity/excess body weight as a predictor of antidepressant efficacy in terms of the degree of improvement in depressive symptoms and rates of response and remission. In addition, we review possible clinical and demographic factors that could modulate this relationship, as well as the possible biological mechanisms underlying the effect of obesity/excess body weight on treatment outcome in depression.

## 2. Search Methods and Selection Criteria

To identify studies on the relationship between obesity/excess body weight and treatment outcome in patients with depressive disorders, we performed a comprehensive literature search using Medline, Embase, and the Cochrane Library on 25 August 2015, using the following search terms: (depression or depressive disorder or depress*), (response or remission or outcome or efficacy or effective* or resistant or resistance depression or depressive), (obesity or body mass index or overweight or BMI or weight), and (antidepressant or antidepress*). We did not filter papers by age/sex or other demographic characteristics. Two independent reviewers (Young Sup Woo and Hye-Jin Seo) determined whether the studies met the selection criteria. Studies were included in our review if they (1) investigated the relationships among depression, treatment outcome, and obesity/excess body weight and (2) were written in English. Studies were excluded if they included only patients with bipolar depression. In addition, the reference lists in the selected articles were searched manually to identify additional references.

## 3. Effects of Body Weight on Changes in Scale Score/Response Rate in Clinical Trials

Table 1 presents a summary of studies that evaluated the relationship between body weight and the response to antidepressants. In 2005, Papakostas *et al.* [10] investigated 369 adult outpatients with major depressive disorder (MDD) and a 17-item Hamilton Depression Rating Scale (HAMD) score of ≥16 at baseline. The subjects were administered open-label, fixed-dose 20 mg fluoxetine for 8 weeks, and those showing a partial or no response to fluoxetine treatment were enrolled in a 4-week, double-blind, randomized study comparing high-dose fluoxetine with augmentation of fluoxetine with desipramine or lithium. They reported the prevalence of overweight (BMI) ≥ 25 kg/m^2^) as 51.4% and that of obesity (BMI ≥ 30 kg/m^2^) as 20.0% at baseline. In their study, greater relative body weight (BMI as a continuous variable) increased risk of non-response (*p* = 0.049, 95% confidence interval (CI) = 1.00–1.08), but the presence of obesity was not significantly associated with non-response to treatment (*p* = 0.160), and the presence of overweight showed trend-level significance (*p* = 0.067). The predictive value of BMI for the response to antidepressants was supported by Khan *et al.* [11]. They evaluated antidepressant responses in 274 adult outpatients with moderate to severe unipolar depression participating in one of 15 randomized, double-blind, placebo-controlled, phase II–IV clinical trials that used selective serotonin reuptake inhibitors (SSRIs; citalopram, escitalopram, fluoxetine, paroxetine, or sertraline) or placebo. Because there was no difference in the mean change in the HAMD-17 score between the normal and overweight groups, the patients were divided into the obese (BMI ≥ 30 kg/m^2^) and non-obese groups. In their study, with an obesity prevalence rate of 33.9%, when adjusting for baseline severity, non-obese patients showed a greater change in the HAMD-17 score than did obese patients. There was a significant effect of BMI on HAMD-17 score change (*p* = 0.018). The effect of obesity on the Montgomery–Åsberg Depression Rating Scale (MADRS) score was not statistically significant (*p* = 0.075).

In a large study designed to discover biomarkers and genotypes predictive of clinical outcome in MDD patients, Kloiber *et al.* [12] reported a slower clinical response in patients with a high BMI (>25 kg/m^2^). In their study, 320 inpatients with MDD were divided into a normal-BMI group (≤25 kg/m^2^, *n* = 173, 54.1%) and a high-BMI group (>25 kg/m^2^, *n* = 147, 45.9%). Various antidepressants were administered at the discretion of the attending physician. The high-BMI group showed a significantly slower response to antidepressant treatment (*p* < 0.01) over five weeks. The high-BMI group was divided into overweight (25 kg/m^2^ < BMI ≤ 30 kg/m^2^) and obese (BMI > 30 kg/m^2^) groups, and the slowest improvement was observed in the obese patients (*p* < 0.05). The difference between obese patients and those with a normal BMI was significant (*p* = 0.05), and there was a trend between overweight and normal-weight patients (*p* = 0.07). The response rate on the HAMD-21 after 5 weeks was 50.0% in normal-BMI patients, 46.5% in overweight patients, and 17.4% in obese patients. The obese patients had a >4-fold higher risk of no response than did normal BMI patients (OR = 4.49, 95% CI = 1.48–13.64, *p* < 0.01). The authors reported that reduced improvement during antidepressant treatment in high-BMI patients was accompanied by smaller improvements in attention and hypothalamic-pituitary-adrenal (HPA) axis dysregulation compared with normal-BMI patients. In another study investigating the association of BMI and response to antidepressants in 797 patients with MDD of at least moderate severity, a higher BMI (as a continuous variable) at baseline was a significant predictor of a poorer outcome on the MADRS scale in the whole sample (*p* = 0.025), and remained significant after controlling for sex, age, and treatment with escitalopram or nortriptyline (*p* = 0.046) [18]. In addition to BMI, obesity (BMI > 30 kg/m^2^) predicted worse outcome on MADRS (*p* = 0.022).

Poor treatment outcome in obese patients with depression was also previously reported in a meta-analysis. Oskooilar *et al.* [13] performed a meta-analysis on data from three completed clinical trials of MDD patients using an 8-week, double-blind, active-controlled comparison design. The participants were adult outpatients (*n* = 59) treated with SSRIs or serotonin and norepinephrine reuptake inhibitors (SNRIs). At baseline, the mean HAMD scores were not significantly different between normal BMI, overweight (25 kg/m^2^ ≤ BMI < 30 kg/m^2^), and obese (BMI ≥ 30 kg/m^2^) groups. After 8-week antidepressant treatment, the changes in scores for all depressive symptom measures (HAMD, MADRS, Clinical Global Impression-Severity (CGI-S)) were significantly different in the normal BMI group compared with the overweight and obese groups. The results of the overweight and obese groups were not significantly different from each other.

The negative effects of obesity/excess body weight on treatment response were also observed in a long-term study. Dennehy *et al.* [14] analyzed data from a cohort of subjects with depressive disorders over a 6-month follow-up. They applied a definition of “sustained remission” in that subjects with a Quick Inventory of Depressive Symptomatology-Self-Rated (QIDS-SR) score of ≤5 at both the initial and 6-month survey assessment were considered to be in sustained remission from depression. Of the 640 patients included in the study population, 140 (21.9%) were in sustained remission, and 348 (54.4%) had not achieved remission at either the initial or 6-month visit. Comparing these two groups of patients, non-remitters had a higher mean BMI (29.8 ± 8.5 kg/m^2^ *vs.* 27.3 ± 6.4 kg/m^2^, respectively, *p* < 0.001) and were more likely to be obese (11.8% *vs.* 4.3%, respectively, *p* = 0.011) compared with the remitters.

**Table 1:** Characteristics of prior studies investigating the associations between depressive disorders, obesity/excess body weight, and antidepressant responses.

Reference	Study Design	Inclusion Criteria	Sample Composition	Administered ADs	Definition and Prevalence of Obesity/Excess Body Weight	Main Treatment Outcome Measure	Main Outcome	Note
Papakostas *et al.* [10]	8-week, open label, non-comparative, fixed dose	DSM-III-R MDD, outpatient, HAMD-17 ≥ 16	Mean age = 39.8 ± 10.4, women = 53.9%	FLX	Overweight (BMI ≥ 25) = 51.4%, Obesity (BMI ≥ 30) = 20.1%	HAMD-17 response	Higher BMI was predictive of poor outcome. Obesity was not predictive of poor outcome.	^–^
Khan *et al.* [11]	Pooled analysis of 15 randomized, double-blind, placebo-controlled trial	DSM-IV MDD, outpatient, moderate to severe	Mean age = 47.2 ± 20.0 (non-obese men), 39.8 ± 12.1 (obese men), 44.6 ± 14.7 (non-obese women), 40.4 ± 16.4 (obese women), women = 54.7%	SSRIs and placebo	Obesity (BMI ≥ 30) = 33.9%	HAMD-17 and MADRS score change	Obesity was predictive of poor outcome in males. Obesity was not predictive of poor outcome in females.	^–^
Kloiber *et al.* [12]	5-week, naturalistic pharmacogenetic study	DSM-IV MDD, inpatient	Mean age = 47.8 ± 14.3, women = 55.3%	Various ADs	Overweight (30 ≥ BMI > 25) = 37.0%, Obesity (BMI > 30) = 10.0%	HAMD-21 response	High BMI (overweight and obesity) was predictive of poor outcome (slower response). Obesity was predictive of poor outcome (non-response).	Munich Antidepressant Response Signature Project [20].
Uher *et al.* [18]	12-week, open-label, part-randomized trial	DSM-IV MDD, at least moderate severity	Mean age = 42.8 ± 11.6 (escitalopram group), 42.7 ± 11.8 (nortriptyline group), Women = 61.0% (escitalopram group), 64.0% (nortriptyline group)	SCIT, NTP	Obesity (BMI ≥ 30) = 14.4%, Overweight (30 > BMI > 25) = 33.9%	MADRS score change	Higher BMI and obesity were predictive of poor outcome. The association between higher BMI/obesity and poor outcome was significant in nortriptyline-treated patients but not in escitalopram-treated patients.	The Genome Based Therapeutic Drugs for Depression (GENDEP) [21].
Sagud *et al.* [15]	Cross-sectional study	DSM-IV MDD, inpatient	Mean age = 49.3 ± 9.7 (non-TRD group), 57.2 ± 10.3 (TRD group)	Various ADs, mostly SSRIs and SNRIs	High BMI (BMI ≥ 27.5) = 46.7%, Central obesity (waist circumference ≥102 cm in men, ≥88 cm in women) = 33.0%	TRD by HAMD-17	High BMI and central obesity were not associated with poor outcome.	TRD: failure to achieve ≥50% reduction after ≥2 antidepressant therapies over ≥8 weeks
Oskooilar *et al.* [13]	Meta-analysis of 3 clinical trials	DSM-IV-TR MDD, outpatient	Age range = 18–65	SSRIs and SNRIs	Overweight (30 > BMI ≥ 25) = 32.2%, Obesity (BMI ≥ 30) = 35.6%	HAMD, MADRS, CGI-S change	Overweight and obesity were predictive of poor outcome.	Clinical trials with double-blind, active controlled design
Toups *et al.* [16]	12-week, randomized trial (with subsequent 16-week extension)	DSM-IV-TR MDD, chronic or recurrent depression, HAMD-17 ≥ 16	Mean age = 38.6 ± 12.8 (normal weight), 44.6 ± 13.0 (overweight), 44.4 ± 12.8 (obesity 1), 43.3 ± 12.7 (obesity 2+), Women = 68.0% (normal weight), 59.9% (overweight), 69.9% (obesity 1), 75.7% (obesity 2+)	SSRI+placebo, SCIT+BUP SR, VEN XR+MIR	Overweight (30 > BMI ≥ 25) = 28.2%, Obesity I (35 > BMI ≥ 30) = 20.1%, Obesity 2+ (BMI ≥ 35) = 26.1%	QIDS-SR remission and response	BMI class was not associated with poor outcome.	Combining Medications to Enhance Depression Outcomes (COMED) [22]
Vogelzangs *et al.* [17]	Naturalistic cohort study with 2-year follow-up	DSM-IV MDD or dysthymia	Mean age = 42.8 ± 11.3, Women = 63.8%	SSRIs, SNRIs, TCAs, TeCAs	Abdominal obesity (waist circumference >88 cm in women, 102 cm in men) = 42.3% (remitted disorder), 40.7% (chronic disorder)	Remission	Abdominal obesity was not associated with poor outcome.	The Netherlands Study of Depression and Anxiety (NESDA) [23].
Dennehy *et al.* [14]	Retrospective/prospective fixed cohort repeated measures design	ICD-9-CM, depression	Mean age = 48.8 ± 11.4 (remission), 48.3 ± 10.9 (non-remission), Women = 82.1% (remission) 82.2% (non-remission)	Various ADs, mostly SSRIs and SNRIs	Obesity (BMI ≥ 30) = 9.6%	Sustained remission	Obesity and higher BMI were associated with poor outcome (non-remission).	The Comorbidities and Symptoms of Depression (CODE) study. Data from initial and six-month surveys

ADs: antidepressants; DSM: Diagnostic and Statistical Manual of Mental Disorders; MDD: Major Depressive Disorder; HAMD: Hamilton Depression Rating Scale; FLX: fluoxetine; BMI: Body Mass Index; SSRI: Selective Serotonin Reuptake Inhibitor; MADRS: Montgomery-Åsberg Depression Rating Scale; SCIT: escitalopram; NTP: nortriptyline; TRD: treatment resistant depression; SNRI: Serotonin and Norepinephrine Reuptake Inhibitor; CGI-S: Clinical Global Impression-Severity; BUP: bupropion; VEN: venlafaxine; MIR: mirtazapine; QIDS-SR: Quick Inventory of Depressive Symptomatology-Self Rated; TCA: Tricyclic Antidepressant; TeCA: Tetracyclic Antidepressant; ICD-9-CM: International Classification of Diseases; 9th Revision; Clinical Modification.

Preliminary results obtained by our group (unpublished) also supported the long-term association of treatment-resistant depression (TRD) and baseline obesity. In that study, we analyzed data from a nationwide prospective study of 541 Korean patients with depressive disorders. For 52 weeks, the subjects were treated with various antidepressants according to the decision of the attending clinician. Baseline obesity was defined as a BMI ≥ 25 kg/m^2^. During the 52 weeks, TRD patients (obesity prevalence = 32.0%) were 1.6-fold more likely to be obese than were non-TRD patients (obesity prevalence = 21.5%, 95% CI = 1.02–2.35, *p* = 0.039).

However, not all studies support a negative effect of body weight on antidepressant responses. Sagud *et al.* [15] reported that a high BMI (≥27.5 kg/m^2^) and central obesity (waist circumference ≥102 cm in men, ≥88 cm in women) were not associated with treatment resistance in MDD. In their study investigating the relationship between TRD and metabolic syndrome and its components in 203 patients with MDD, TRD was observed in 26.1% of patients, and metabolic syndrome was observed in 33.5% of patients. The prevalence rates of high BMI in TRD and non-TRD patients were 45.3% and 47.3%, respectively (*p* = 0.923). The prevalence of central obesity was not significantly different between TRD (37.7%) and non-TRD (31.3%) patients (*p* = 0.495). These results were not changed after adjusting for sex and age. The odds ratio of treatment resistance for high BMI *vs.* normal BMI was 0.935 (95% CI = 0.419–2.089), and that for central obesity was 0.983 (95% CI = 0.466–2.072).

Toups *et al.* [16] also investigated the moderating effect of obesity on antidepressant treatment outcomes in 662 chronic or recurrent MDD patients treated with escitalopram, bupropion, venlafaxine, mirtazapine, or combinations of these antidepressants. Subjects were categorized into 4 groups for the analysis; normal or underweight (BMI < 25 kg/m^2^), overweight (BMI 25–29 kg/m^2^), obese I (BMI 30–34 kg/m^2^), and obese II+ (BMI ≥ 35 kg/m^2^). The authors did not find any differences in antidepressant treatment outcomes across the BMI classes. In week 12, there were no significant differences among the BMI groups with respect to overall outcome, measured as QIDS-SR remission or response. The frequency, intensity, and burden of side effects were also not significantly different across groups in week 12, after adjustment. In week 28, there were no differences in depression outcome or functional measures among groups.

Furthermore, in a recent study regarding the effects of metabolic/inflammatory dysregulation on an antidepressant treatment course [17], abdominal obesity (waist circumference >88/102 cm (women/men)) was not associated with a 2-year chronicity of depressive disorders, which was indicative of no response to antidepressants. The prevalence of abdominal obesity was 42.3% in patients achieving remission and 40.7% in chronic patients (*p* = 0.770). After adjusting for age, sex, and other confounding variables, the association of abdominal obesity with depression chronicity was not significant (OR = 0.92, 95% CI = 0.56–1.52). Rather, significant predictors of depression chronicity were elevated: interleukin-6 (IL-6), low high-density lipoprotein (HDL)-cholesterol, hypertriglyceridemia, and hyperglycemia. These results indicated that a certain degree of inflammatory and metabolic dysregulation, but not abdominal obesity, could worsen the course of depression due to a reduced antidepressant treatment response.

## 4. Possible Explanations for the Variability in Results among Studies

The discrepancies in the results of the above studies investigating the relationships between obesity/excess body weight and antidepressant responses could be attributed to sample composition (e.g., age and sex of the patients), heterogeneity among depressive symptoms, other comorbid medical conditions, or differences in the antidepressants administered.

### 4.1. Atypical Depressive Symptoms

A general consensus is that clinical heterogeneity complicates efforts to identify the biological, genetic, and environmental underpinnings of depression [24]. Especially, the atypical subtype is thought to contribute to the variability in associations with biological measures. Patients with atypical subtype depression tend to have a higher prevalence of metabolic syndrome, and in particular its obesity-related disturbances [25], and show higher inflammatory levels of inflammatory markers [20,21].

Hence, the differential effect of obesity/excess body weight on improvement of atypical depressive symptoms could provide insight into the relationship between body weight and antidepressant treatment outcome. Among the studies discussed above, three included measures for atypical symptoms. Kloiber *et al.* [12] reported a correlation between high BMI and atypicality at baseline. A higher baseline BMI was associated with a significantly lower score on the HAMD subscale for neurovegetative symptoms (non-atypical subscale; loss of weight, insomnia, and loss of appetite) but did not differ in the remaining 16 items at baseline. Consistent with this result, Toups *et al.* [16] reported that atypical features were more common in high-BMI subjects, and melancholic features were more common in low-BMI subjects, consistent with the diagnostic criteria of increased and decreased appetite, respectively, for these subtypes. With increasing BMI, the rate of subjects with atypical features increased (*p* = 0.037), but the rate of melancholic features decreased (*p* = 0.028).

The discrepancies among the results of the above studies may be related to the effects of atypical features on the association between obesity and antidepressant responses. According to Uher *et al.* [18], the moderation of the response to antidepressants by BMI was relatively specific to neurovegetative symptoms. The high-BMI patients showed less improvement in the neurovegetative symptoms of sleep and appetite (*p* < 0.001). However, baseline BMI did not significantly predict changes in mood or cognitive symptoms. With regard to continuous BMI measurements, the moderating effect of obesity strongly influenced changes in neurovegetative symptoms, modestly but significantly influenced observed mood (*p* = 0.031), and weakly influenced cognitive symptoms (*p* = 0.058). The authors suggested that these observations indicated the moderating effect of body weight on antidepressant responses is relatively specific to neurovegetative symptoms [18]. However, atypical features did not affect the negative association between obesity and antidepressant responses in a study by Toups *et al.* [16]. It should be taken into consideration that there are two major elements of atypical depression, *i.e.*, reverse neurovegetative symptoms and reactivity of mood [22]. Benazzi [23] proposed the exclusion of mood reactivity from the atypical feature specifiers for unipolar depression. It is possible that neurovegetative symptoms, and not mood reactivity, have a moderating effect on the relationship between obesity and antidepressant responses.

Overall, although there are still insufficient results to allow definitive conclusions to be drawn, the association of atypical features and obesity/excess body weight could be regarded as supported [26,27], and the moderating effect of atypical features on antidepressant responses by obesity/excess body weight was also supported. Furthermore, the “cytokine hypothesis” of depression could be extended from the development of depression [28] to its clinical course, based on overlapping findings among atypical features (especially neurovegetative symptoms), poor treatment outcome, and obesity in inflammatory and metabolic components related to inflammation [17,21,29].

### 4.2. Sex Differences

The results of epidemiological studies indicated the effects of sex on the association between body weight and treatment outcome [30,31]. In a study by Khan *et al.* [11], obesity was associated with poor antidepressant responses in men, whereas women showed significant improvement regardless of BMI category. Obese males treated with antidepressants showed no significant improvement in the HAMD-17 or MADRS score compared with the changes seen in the placebo group. Females in both BMI categories (obese and non-obese) and non-obese male subjects treated with SSRIs showed a significant improvement in HAMD-17 and MADRS scores compared with subjects assigned a placebo. However, in another study that reported an association between worse treatment outcome and obesity [18], sex had no effects on the overall response to antidepressants or the moderation of antidepressant responses by body weight (*p* = 0.317). However, the role of sex in the association between obesity and poor improvement was significant when neurovegetative symptoms were considered as an outcome. The effects of obesity on changes in neurovegetative symptoms were strongest among men treated with nortriptyline (*p* = 0.005) and moderate in women treated with either nortriptyline (*p* = 0.020) or escitalopram (*p* = 0.013). The effect was not significant among escitalopram-treated men (*p* = 0.482).

In our preliminary results (unpublished), TRD was associated with male sex (OR = 1.82, 95% CI = 1.20–2.75, *p* = 0.005). When we stratified the subjects into three groups, *i.e.*, males, premenopausal females, and post-menopausal females, obesity predicted treatment resistance only in post-menopausal female TRD patients (OR = 2.41, 95% CI = 1.25–4.66, *p* = 0.009). The effect of obesity on treatment resistance was not significant in males or premenopausal females. In male subjects, the presence of one or more of hypertension, hyperglycemia, and hypercholesterolemia predicted TRD (OR = 2.32).

The three-way interaction among sex, obesity/excess body weight, and treatment response should be investigated in detail in future studies. Although there are supporting results, the directions of the findings are inconsistent. A poor response has been associated with obesity in men treated with serotonergic antidepressants [11] and noradrenergic antidepressants, but not in either sex treated with serotonergic antidepressants [18] or post-menopausal women treated primarily with SSRIs or SNRIs in our preliminary experiments.

The different responses may be the result of differential body fat percentage and distribution occurring as a function of obesity and sex, rather than overall body weight [32]. There is increasing evidence suggesting that visceral adipose tissue is an important source of inflammatory adipocytokines (e.g., tumor necrosis factor-α (TNF-α), IL-6, plasminogen activator inhibitor-1, IL-6, and angiotensinogen) and hormones (e.g., leptin, adiponectin, and resistin) [33,34,35]. Visceral adipose tissue from obese subjects has also been shown to secrete higher levels of these proinflammatory adipocytokines compared with non-obese subjects [36,37,38]. In a recent meta-analysis [39], patients with higher levels of inflammation at baseline responded less well to subsequent treatment, although this finding was not statistically significant. Moreover, a significant decrease in TNF-α levels was observed only in treatment responders, whereas treatment resistance was associated with persistently elevated TNF-α levels. Estrogen may protect against this increase in inflammation in treatment non-responsive depressive disorders. Studies in postmenopausal women have shown a reversal of increased adipocytokines caused by increased visceral adiposity during menopause [40] with repletion of estrogen [41,42] and a specific anti-inflammatory action of estrogen on the brain [43,44]. Moreover, estrogen decreases neuroinflammation by modulating microglial activity [43].

In addition, the role of estrogen should be considered as a modulating factor of sex differences in treatment outcome to different classes of antidepressants in obese patients. The results of several studies [45,46,47,48,49], but not all [50,51], support a role of estrogen in modulating serotonergic antidepressant responses. Premenopausal women are more responsive to serotonergic antidepressants than norepinephrinergic tetracyclic antidepressants [46]. The literature includes evidence supporting the enhanced role of estrogen in the serotonergic system [52,53,54,55,56]. In preclinical and clinical studies, estrogen administration enhanced the antidepressant effect of SSRIs [57,58,59].

Among three studies reporting negative associations between obesity and antidepressant responses, two [16,17] included information regarding the influence of age and sex. In these two studies, the proportion of female subjects was relatively high (63.8%–68.1%) compared with studies reporting positive associations (53.9%–62.4%), with the exception of one study by Dennehy [14] that reported an association between non-remission and obesity without adjusting for sex differences. Since the mean age at menopause is approximately 50 years [60], and the mean age of the subjects in the abovementioned studies was early 40s, it is possible that a large portion of subjects were premenopausal women, which may have increased the likelihood of a negative association between obesity and a poor response to antidepressants.

### 4.3. Medical Comorbidities

Interestingly, all three studies that reported negative associations between obesity and the response to antidepressants assessed the presence of comorbid medical conditions, while none of those with positive results did. The poor antidepressant treatment outcome of depressive disorders was associated with higher glucose levels [15], low HDL-cholesterol levels, hypertriglyceridemia, and hyperglycemia [17]. However, as the lack of an association between obesity and poor treatment outcome was observed upon univariate analysis before adjusting for comorbid conditions, this could be merely a coincidence. Future studies should therefore include medical comorbidities as covariates.

### 4.4. Class of Antidepressants

In one study investigating the association of BMI and the response to antidepressants, BMI differentially predicted a poor response to escitalopram, a serotonin reuptake inhibitor and nortriptyline, a noradrenaline reuptake inhibitor [18]. In that study by Uher *et al.*, there was a significant effect of baseline BMI among nortriptyline-treated (*p* = 0.003), but not escitalopram-treated, subjects (*p* = 0.547). In addition to BMI, obesity (BMI > 30 kg/m^2^) also predicted a worse outcome in the MADRS total score change among nortriptyline-treated (*p* = 0.001), but not escitalopram-treated, subjects.

In contrast to the above results, a study of adolescent TRD subjects [61] did not support different effects of BMI on treatment response to different antidepressant classes. With respect to treatment course, rates of remission at 24 weeks were unrelated to BMI in univariate analyses (*p* = 0.77) and after adjustment for baseline differences in age and drug- and alcohol-related impairments (overweight *vs.* normal weight: OR = 1.15, 95% CI = 0.58–2.27, *p* = 0.70; obese *vs.* normal weight: OR = 0.88, 95% CI = 0.50–1.55, *p* = 0.65). No moderating effect of BMI on treatment outcome (medication, *p* = 0.82; cognitive behavior therapy, *p* = 0.64) on remission was detected.

As the subjects of most studies included in the present review were treated with various antidepressants, it is difficult to interpret the effects of antidepressant classes. The effects of antidepressants with different modes of action on treatment response in patients with obesity should be investigated in future studies.

## 5. Mechanisms Underlying the Association between Obesity and Antidepressant Responses

As shown in a recent review by Mansur *et al.* [19], obesity is closely related to depressive disorders. However, the nature of this association remains poorly understood [19], and the mechanisms underlying the potential association between obesity and treatment outcome to antidepressant treatment have not been fully established.

### 5.1. Phosphoinositide 3-Kinase (PI3K)/Protein Kinase B (Akt)/Glycogen Synthase Kinase (GSK)-3β Pathway Regulation By Adipocytokines

One of the suggested mechanisms to account for the possible association between obesity and antidepressant responses in depressed patients is insulin resistance. Brain insulin resistance implies that an increase in insulin levels does not induce an increased neuronal activity in obese individuals in contrast to lean individuals [62]. Brain insulin resistance caused by obesity is associated with alterations in neurocircuits and brain regions suggested to be involved in affective disorders [63], including the prefrontal cortex [64,65], amygdala/hippocampus [65], anterior cingulate cortex [66], and ventral striatum [65]. Recent studies suggested that these brain regions may also be related to antidepressant responses [67,68,69,70], and some of these brain regions overlapped with those showing a high insulin receptor density and mRNA expression levels [71,72,73,74]. In these regions, insulin inhibits the reuptake of norepinephrine, and neuronal firing modulates norepinephrine and dopamine transporter mRNA concentration and catecholamine turnover and stimulates phosphoinositol turnover [74,75,76,77,78]. Furthermore, insulin disturbance may affect the metabolism of brain monoamines and expression of serotonergic receptors in diabetic animal models and humans [79,80,81,82,83]. The association between insulin signal impairment and antidepressant effects can be explained by the role of insulin signaling molecules, including glycogen synthase (GSK)-3β, mammalian target of rapamycin (mTOR), protein kinase B (Akt), and extracellular signal-regulated kinase (ERK) 1/2 [84]. The phosphoinositide 3-kinase (PI3K)/Akt pathway and its components, including GSK-3β and mTOR, have been implicated in the response to antidepressants [85].

### 5.2. Inflammatory Dysregulation

The most likely mechanism connecting obesity to antidepressant responses in depressive disorders is inflammatory dysregulation, which has been recognized as a core feature of obesity and has also been shown to be involved in depressive disorders [19]. Obesity is now considered to be a persistent, low-grade, proinflammatory state [86]. Adipose tissue, as an active endocrine organ, secretes proinflammatory factors, including IL-1, IL-6, TNF-α, and C-reactive protein [87]. In addition, adipose tissue macrophages may be a source of proinflammatory factors and can regulate the secretory activity of adipocytes [88]. Peripheral proinflammatory cytokines cross the blood–brain barrier (BBB) via specific transporters [89]. In addition, peripheral inflammatory mediators may enter the brain through somewhat compromised sites of the BBB, *i.e.*, at circumventricular organs [90]. Local release of cytokines can also stimulate peripheral afferent nerve fibers that innervate peripheral tissues, such as the vagus nerve, ultimately activate microglia to produce cytokines in the brain [91]. Obesity may negatively affect prognosis and outcome in depressive disorders through various inflammatory processes [92,93,94,95,96]. Several studies have shown that high levels of proinflammatory cytokines are associated with a reduced responsiveness to antidepressant therapy [97,98,99,100] and increased severity of depression [100,101,102].

The regulation of the PI3K/Akt/GSK-3β pathway may also be involved in the associations between obesity-induced inflammation and antidepressant responses. Brain IL-1β and TNF-α signaling result in subsequent activation of c-jun N terminal kinase (JNK), which leads to inhibition of the insulin receptor substrate 1 (IRS1)/PI3K/Akt pathway [103]. The PI3K/Akt signaling pathway in the brain regulates the molecular mechanisms of depression [104]. As PI3K and Akt seem to regulate key inflammatory cytokines resulting in immune cell activation [105], Akt and GSK3 signaling changes may contribute to specific therapeutic effects on depression [106]. Brain intracellular signal transduction systems, including the Akt/GSK3 pathway, have been shown to be altered in patients with psychiatric illness [107]. In addition, both serotonin and dopamine exert their actions, in part, by modulating Akt/GSK3 activity [108]. Proinflammatory cytokines induced by adipose tissue may affect the treatment response of patients with depressive disorders through these signaling pathways.

PI3K pathways regulate metabolism, cell growth, and cell survival [109]. The phospholipid second messengers generated by the PI3Ks provide a common mechanism for multiple steps of intracellular signal transduction [106]. Akt is a major downstream target of the PI3Ks for regulating cell growth and cell migration [110]. Phosphatidylinositol 3,4,5-triphosphate (PIP3), a product of PI3K, binds to Akt and leads to membrane recruitment of Akt. The activation of Akt leads to phosphorylation of GSK3β, which is active in resting cells but is inactivated by phosphorylation [106].

A chronic, low-grade inflammatory state within visceral adipose tissue contributes to insulin resistance through activation of inflammatory signaling cascades within immune cells (e.g., macrophages) [111,112]. The PI3K/Akt/GSK3β pathway is also a key mechanism in the regulation of glucose transport and insulin metabolism in insulin-sensitive peripheral tissues [103,113,114]. The serine/threonine kinase GSK3β plays roles in the regulation of many brain functions, including the control of energy homeostasis and mood [115].

This pathway has emerged as a mediator of antidepressant effects of commonly used psychotropic agents [116]. Antidepressants, such as fluoxetine and imipramine, and lithium were demonstrated to increase Akt activity [117,118,119]. GSK3β has been well characterized as a target of the mood stabilizers lithium and antidepressants, which can directly or indirectly block its actions [120]. The actions of several key neurotransmitter receptors, whose crucial roles in antidepressant mechanisms of action have been well established, were shown to potentiate Akt activity, thus mediating these effects [104]. This was found to be the case for serotonin 1A (5-HT1A) [121] and 5-HT1B [122] receptors, cannabinoid receptor subtype 1 (CB1R) [123], brain-derived neurotropic factor (BDNF) receptor, *i.e.*, tropomyosin receptor kinase B (TrkB) [124,125,126], and the insulin receptor [104,127,128]. Akt is also involved in the regulation of survival transcription factors, such as nuclear factor κB (NF-κB) and cyclic AMP response element binding protein (CREB), as well as those involved in pro-death gene expression, such as the forkhead box O (FOXO) family [129]. For example, the stimulation of 5-HT receptors leads to activation of Akt and subsequent inhibition of GSK3β or transcription factor FOXO activity [104]. These effects were shown to be induced via inactivation of molecules positively correlated with a depressive-like phenotype, such as GSK3β and FOXO3a, by phosphorylation [104]. Overall, antidepressants acting on 5-HT can be considered to activate Akt and inhibit GSK3β. Thus, obesity may attenuate depression treatment responses through induced inflammatory cytokines that alter Akt and GSK3β activity, resulting in changes in several key neurotransmitters.

The mediating role of adipocytokines on antidepressant responses in depressive disorders could be related to diminished levels of BDNF [130]. These cytokines decrease the expression BDNF and CREB and increase the expression of NK-κB, a primary transcription factor that functions in the initiation of inflammatory responses [131]. BDNF binds to the TrkB receptor and activates multiple signaling cascades, such as PI3K/Akt pathway and extracellular signal-regulated kinase 1/2 (ERK1/2) pathway [132], which regulate the transcription factor CREB [133,134]. CREB has been reported as a key mediator of BDNF-induced gene expression and cell survival [135]. Decreased expression of BDNF leads to decreased neuronal repair, decreased neurogenesis, and increased activation of the glutamatergic pathway, which also contributes to neuronal apoptosis [130]. Stimulation of the extra-synaptic *N*-methyl-d-aspartate (NMDA) glutamate receptor not only causes excitotoxic damage to the neurons and astrocytes, but also results in a decrease in synthesis of BDNF [136]. The net result of these changes is loss of astrocytes and oligodendroglia and neuronal apoptosis, particularly in the subgenual prefrontal cortex, amygdala, and hippocampus, brain regions thought to be crucially involved in the genesis of the symptoms of depressive disorder [137].

Disturbances in hippocampal neurogenesis may contribute to the symptoms of depression [138]. The antidepressant effects of electroconvulsive therapy were accompanied by inducing hippocampal neurogenesis in adult non-human primates [139]. Disruption of antidepressant induced hippocampal neurogenesis by selective irradiation of the hippocampus also blocked the antidepressant effects of fluoxetine and imipramine [140], suggesting that neurogenesis is fundamental in determining the functional consequences of antidepressant treatment [138].

Another possible mechanism by which inflammation affects antidepressant responses is serotonin metabolism. The activity of indoleamine 2–3 dioxygenase (IDO), a catabolizing enzyme of tryptophan and serotonin, was elevated in the plasma of depressed patients and enhanced by a proinflammatory cytokines [141]. Activation of this enzyme leads to increased consumption of tryptophan, thus reducing the availability of serotonergic neurotransmission and inducing the production of detrimental tryptophan catabolites with neurotoxic effects [142]. It has also been shown that the activity of the dopaminergic system is reduced in response to inflammation [143], while cytokines enhance the reuptake of monoamine neurotransmitters, thereby reducing their functionally important intrasynaptic concentrations in the brain [144].

Increased levels of the neurotoxic tryptophan catabolite quinolinic acid cause excitotoxic neurodegenerative changes [145]. This neurodegeneration can contribute to changes in brain structure and function and to treatment resistance in depressive disorder [141].

In addition, disturbances in neurotransmitter function can lead to increased mood symptoms and to changes in treatment response to medications targeting monoamine neurotransmitters in depressive disorder [146,147].

### 5.3. Oxidative/Nitrosative Stress

Obesity and depressive disorders share the pathophysiology of increased oxidative and nitrosative stress and reduced antioxidant defenses. Antioxidants are inversely associated with both obesity [148,149,150] and MDD [151], and oxidative/nitrosative stress pathways are involved in treatment resistance and in the mechanisms of action of antidepressants [152]. Moreover, lowered serum levels of antioxidants, such as coenzyme Q10 and zinc, could be biological markers of treatment resistance to antidepressant therapy [153,154]. In the depressed state, compromised antioxidant defenses [155] activate oxidative/nitrosative stress pathways and damage to fatty acids, proteins, DNA and mitochondria, and initiate autoimmune responses [156]. Moreover, damage by oxidative/nitrosative stress and subsequent autoimmune responses are major causes of progression and chronicity in MDD [157,158,159]. Oxidative/nitrosative stress pathway activation could be induced by inflammation, which damages lipids, DNA, and proteins, resulting in mitochondrial dysfunction and increased apoptosis, cell membrane damage, and protein aggregation [160]. TNF-α and IL-1 induce both astrocytes and microglia to release reactive oxygen and nitrogen species that amplify oxidative stress and impair glutamine reuptake, which is especially vulnerable to oxidative damage [161,162]. Reactive oxygen species (ROS) are generated along the electron transport chain as a product of mitochondrial respiration. Under physiological conditions, ROS are detoxified by endogenous antioxidant enzymes (e.g., superoxide dismutase). Under pathogenic conditions, an imbalance occurs between ROS accumulation and elimination, resulting in hazardous effects on lipids, proteins, DNA, and the immune system, e.g., neoepitopes [163]. Correlations have been reported between severity of depression and the magnitude of disturbances in oxidative indices [164]. Andreazza *et al.* [165] found that bipolar disorder is associated with a marked increase in DNA damage in white blood cells due to oxidative stress, and such DNA damage was correlated with the severity of depressive symptoms. Oxidative and nitrosative stress can also be more pronounced in cases of unipolar depression with more severe presentation (e.g., suicidality) [166].

## 6. Summary with Conclusions

In conclusion, accumulating evidence has indicated that the relationship between depressive disorder and obesity is bidirectional rather than a simple comorbidity. In this review, we also discussed the possible relationship between obesity and antidepressant responses in depressive disorders. As obesity is a core feature of metabolic syndrome [167], the concept of “metabolic-mood syndrome” suggested by some authors [74,168,169] could be expanded to include the association between obesity and antidepressant responses.

In summary, the negative impact of obesity on treatment outcomes was suggested by several studies, and it implies that obesity should be considered a predictor of poor treatment response and should be managed together with depressive symptoms. It is likely that treatment options targeting mechanisms underlying the associations between obesity and poor antidepressant response will have superior outcomes in obese patients with depressive disorders. Moreover, restrained use of antidepressants with weight-gain potentials in early phase of treatment would be beneficial to patients at risk of obesity or related morbidities. It is also noteworthy that male sex and post-menopausal state or atypical features of depression might increase the risk of poor treatment outcomes associated with obesity.

However, some studies have suggested possible confounding effects of sex, age, menopausal status, psychopathology, other medical comorbidities, and antidepressants. Moreover, most studies did not analyze the mechanisms underlying the association between obesity and antidepressant responses. For example, evidence suggests that neurocognitive dysfunction, considered a core feature of mood disorders, is more prominent in overweight/obese individuals with mood disorders [170]. Furthermore, elevation of inflammatory cytokine levels could induce cognitive and affective dysfunction [171]. Therefore, further studies are required to characterize the interactions between obesity and antidepressant responses in more homogenous populations with potential mediators, including cognitive measures and inflammatory markers. Such investigations would yield an improved understanding of the mechanisms underlying the effects of antidepressants on depressive disorders, potentially leading to novel treatments that could improve the treatment outcomes for depressive disorders.

## References

[1] Nelson J.C., Papakostas G.I. (2009). Atypical antipsychotic augmentation in major depressive disorder: A meta-analysis of placebo-controlled randomized trials. Am. J. Psychiatry.

[2] Rush A.J., Trivedi M.H., Wisniewski S.R., Nierenberg A.A., Stewart J.W., Warden D., Niederehe G., Thase M.E., Lavori P.W., Lebowitz B.D. (2006). Acute and longer-term outcomes in depressed outpatients requiring one or several treatment steps: A STAR*D report. Am. J. Psychiatry.

[3] Vieta E., Colom F. (2011). Therapeutic options in treatment-resistant depression. Ann. Med..

[4] Turner P., Kantaria R., Young A.H. (2014). A systematic review and meta-analysis of the evidence base for add-on treatment for patients with major depressive disorder who have not responded to antidepressant treatment: A european perspective. J. Psychopharmacol..

[5] Nierenberg A.A. (2003). Predictors of response to antidepressants general principles and clinical implications. Psychiatr. Clin. N. Am..

[6] Souery D., Oswald P., Massat I., Bailer U., Bollen J., Demyttenaere K., Kasper S., Lecrubier Y., Montgomery S., Serretti A. (2007). Clinical factors associated with treatment resistance in major depressive disorder: Results from a european multicenter study. J. Clin. Psychiatry.

[7] Dudek D., Rybakowski J.K., Siwek M., Pawlowski T., Lojko D., Roczen R., Kiejna A. (2010). Risk factors of treatment resistance in major depression: Association with bipolarity. J. Affect. Disord..

[8] Bennabi D., Aouizerate B., El-Hage W., Doumy O., Moliere F., Courtet P., Nieto I., Bellivier F., Bubrovsky M., Vaiva G. (2015). Risk factors for treatment resistance in unipolar depression: A systematic review. J. Affect. Disord..

[9] Toups M.S., Trivedi M.H. (2011). Role of metabolic dysfunction in treatment resistance of major depressive disorder. Neuropsychiatry.

[10] Papakostas G.I., Petersen T., Iosifescu D.V., Burns A.M., Nierenberg A.A., Alpert J.E., Rosenbaum J.F., Fava M. (2005). Obesity among outpatients with major depressive disorder. Int. J. Neuropsychopharmacol..

[11] Khan A., Schwartz K.A., Kolts R.L., Brown W.A. (2007). BMI, sex, and antidepressant response. J. Affect. Disord..

[12] Kloiber S., Ising M., Reppermund S., Horstmann S., Dose T., Majer M., Zihl J., Pfister H., Unschuld P.G., Holsboer F. (2007). Overweight and obesity affect treatment response in major depression. Biol. Psychiatry.

[13] Oskooilar N., Wilcox C.S., Tong M.L., Grosz D.E. (2009). Body mass index and response to antidepressants in depressed research subjects. J. Clin. Psychiatry.

[14] Dennehy E.B., Robinson R.L., Stephenson J.J., Faries D., Grabner M., Palli S.R., Stauffer V.L., Marangell L.B. (2015). Impact of non-remission of depression on costs and resource utilization: From the comorbidities and symptoms of depression (CODE) study. Curr. Med. Res. Opin..

[15] Sagud M., Mihaljevic-Peles A., Uzun S., Cusa B.V., Kozumplik O., Kudlek-Mikulic S., Mustapic M., Barisic I., Muck-Seler D., Pivac N. (2013). The lack of association between components of metabolic syndrome and treatment resistance in depression. Psychopharmacology.

[16] Toups M.S., Myers A.K., Wisniewski S.R., Kurian B., Morris D.W., Rush A.J., Fava M., Trivedi M.H. (2013). Relationship between obesity and depression: Characteristics and treatment outcomes with antidepressant medication. Psychosom. Med..

[17] Vogelzangs N., Beekman A.T., van Reedt Dortland A.K., Schoevers R.A., Giltay E.J., de Jonge P., Penninx B.W. (2014). Inflammatory and metabolic dysregulation and the 2-year course of depressive disorders in antidepressant users. Neuropsychopharmacology.

[18] Uher R., Mors O., Hauser J., Rietschel M., Maier W., Kozel D., Henigsberg N., Souery D., Placentino A., Perroud N. (2009). Body weight as a predictor of antidepressant efficacy in the gendep project. J. Affect. Disord..

[19] Mansur R.B., Brietzke E., McIntyre R.S. (2015). Is there a “metabolic-mood syndrome”? A review of the relationship between obesity and mood disorders. Neurosci. Biobehav. Rev..

[20] Kaestner F., Hettich M., Peters M., Sibrowski W., Hetzel G., Ponath G., Arolt V., Cassens U., Rothermundt M. (2005). Different activation patterns of proinflammatory cytokines in melancholic and non-melancholic major depression are associated with HPA axis activity. J. Affect. Disord..

[21] Lamers F., Vogelzangs N., Merikangas K.R., de Jonge P., Beekman A.T., Penninx B.W. (2013). Evidence for a differential role of HPA-axis function, inflammation and metabolic syndrome in melancholic *vs.* atypical depression. Mol. Psychiatry.

[22] Angst J., Gamma A., Sellaro R., Zhang H., Merikangas K. (2002). Toward validation of atypical depression in the community: Results of the zurich cohort study. J. Affect. Disord..

[23] Benazzi F. (2002). Should mood reactivity be included in the DSM-IV atypical features specifier?. Eur. Arch. Psychiatry Clin. Neurosci..

[24] Penninx B.W., Milaneschi Y., Lamers F., Vogelzangs N. (2013). Understanding the somatic consequences of depression: Biological mechanisms and the role of depression symptom profile. BMC Med..

[25] Lamers F., de Jonge P., Nolen W.A., Smit J.H., Zitman F.G., Beekman A.T., Penninx B.W. (2010). Identifying depressive subtypes in a large cohort study: Results from the netherlands study of depression and anxiety (NESDA). J. Clin. Psychiatry.

[26] Hasler G., Pine D.S., Gamma A., Milos G., Ajdacic V., Eich D., Rossler W., Angst J. (2004). The associations between psychopathology and being overweight: A 20-year prospective study. Psychol. Med..

[27] Kendler K.S., Eaves L.J., Walters E.E., Neale M.C., Heath A.C., Kessler R.C. (1996). The identification and validation of distinct depressive syndromes in a population-based sample of female twins. Arch. Gen. Psychiatry.

[28] Raedler T.J. (2011). Inflammatory mechanisms in major depressive disorder. Curr. Opin. Psychiatry.

[29] Liu C.S., Carvalho A.F., McIntyre R.S. (2014). Towards a “metabolic” subtype of major depressive disorder: Shared pathophysiological mechanisms may contribute to cognitive dysfunction. CNS Neurol. Disord. Drug Targets.

[30] Blaine B. (2008). Does depression cause obesity?: A meta-analysis of longitudinal studies of depression and weight control. J. Health Psychol..

[31] Lim W., Thomas K.S., Bardwell W.A., Dimsdale J.E. (2008). Which measures of obesity are related to depressive symptoms and in whom?. Psychosomatics.

[32] Keers R., Aitchison K.J. (2010). Gender differences in antidepressant drug response. Int. Rev. Psychiatry.

[33] Tilg H., Moschen A.R. (2006). Adipocytokines: Mediators linking adipose tissue, inflammation and immunity. Nat. Rev. Immunol..

[34] Berg A.H., Scherer P.E. (2005). Adipose tissue, inflammation, and cardiovascular disease. Circ. Res..

[35] Gustafson B., Hammarstedt A., Andersson C.X., Smith U. (2007). Inflamed adipose tissue: A culprit underlying the metabolic syndrome and atherosclerosis. Arterioscler. Thromb. Vasc. Biol..

[36] He G., Pedersen S.B., Bruun J.M., Lihn A.S., Jensen P.F., Richelsen B. (2003). Differences in plasminogen activator inhibitor 1 in subcutaneous *vs.* omental adipose tissue in non-obese and obese subjects. Horm. Metab. Res..

[37] Winkler G., Kiss S., Keszthelyi L., Sapi Z., Ory I., Salamon F., Kovacs M., Vargha P., Szekeres O., Speer G. (2003). Expression of tumor necrosis factor (TNF)-α protein in the subcutaneous and visceral adipose tissue in correlation with adipocyte cell volume, serum TNF-α, soluble serum TNF-receptor-2 concentrations and c-peptide level. Eur. J. Endocrinol..

[38] Maury E., Ehala-Aleksejev K., Guiot Y., Detry R., Vandenhooft A., Brichard S.M. (2007). Adipokines oversecreted by omental adipose tissue in human obesity. Am. J. Physiol. Endocrinol. Metab..

[39] Strawbridge R., Arnone D., Danese A., Papadopoulos A., Herane Vives A., Cleare A.J. (2015). Inflammation and clinical response to treatment in depression: A meta-analysis. Eur. Neuropsychopharmacol..

[40] Carr M.C. (2003). The emergence of the metabolic syndrome with menopause. J. Clin. Endocrinol. Metab..

[41] Zegura B., Guzic-Salobir B., Sebestjen M., Keber I. (2006). The effect of various menopausal hormone therapies on markers of inflammation, coagulation, fibrinolysis, lipids, and lipoproteins in healthy postmenopausal women. Menopause.

[42] Mueck A.O., Genazzani A.R., Samsioe G., Vukovic-Wysocki I., Seeger H. (2007). Low-dose continuous combinations of hormone therapy and biochemical surrogate markers for vascular tone and inflammation: Transdermal *vs.* oral application. Menopause.

[43] Vegeto E., Benedusi V., Maggi A. (2008). Estrogen anti-inflammatory activity in brain: A therapeutic opportunity for menopause and neurodegenerative diseases. Front. Neuroendocrinol..

[44] Dheen S.T., Kaur C., Ling E.A. (2007). Microglial activation and its implications in the brain diseases. Curr. Med. Chem..

[45] Kornstein S.G., Schatzberg A.F., Thase M.E., Yonkers K.A., McCullough J.P., Keitner G.I., Gelenberg A.J., Davis S.M., Harrison W.M., Keller M.B. (2000). Gender differences in treatment response to sertraline *vs.* imipramine in chronic depression. Am. J. Psychiatry.

[46] Martenyi F., Dossenbach M., Mraz K., Metcalfe S. (2001). Gender differences in the efficacy of fluoxetine and maprotiline in depressed patients: A double-blind trial of antidepressants with serotonergic or norepinephrinergic reuptake inhibition profile. Eur. Neuropsychopharmacol..

[47] Khan A., Brodhead A.E., Schwartz K.A., Kolts R.L., Brown W.A. (2005). Sex differences in antidepressant response in recent antidepressant clinical trials. J. Clin. Psychopharmacol..

[48] Quitkin F.M., Stewart J.W., McGrath P.J., Taylor B.P., Tisminetzky M.S., Petkova E., Chen Y., Ma G., Klein D.F. (2002). Are there differences between women’s and men’s antidepressant responses?. Am. J. Psychiatry.

[49] Young E.A., Kornstein S.G., Marcus S.M., Harvey A.T., Warden D., Wisniewski S.R., Balasubramani G.K., Fava M., Trivedi M.H., John Rush A. (2009). Sex differences in response to citalopram: A STAR*D report. J. Psychiatr. Res..

[50] Parker G., Parker K., Austin M.P., Mitchell P., Brotchie H. (2003). Gender differences in response to differing antidepressant drug classes: Two negative studies. Psychol. Med..

[51] Pinto-Meza A., Usall J., Serrano-Blanco A., Suarez D., Haro J.M. (2006). Gender differences in response to antidepressant treatment prescribed in primary care. Does menopause make a difference?. J. Affect. Disord..

[52] Kendall D.A., Stancel G.M., Enna S.J. (1982). The influence of sex hormones on antidepressant-induced alterations in neurotransmitter receptor binding. J. Neurosci..

[53] Halbreich U., Rojansky N., Palter S., Tworek H., Hissin P., Wang K. (1995). Estrogen augments serotonergic activity in postmenopausal women. Biol. Psychiatry.

[54] Gundlah C., Alves S.E., Clark J.A., Pai L.Y., Schaeffer J.M., Rohrer S.P. (2005). Estrogen receptor-β regulates tryptophan hydroxylase-1 expression in the murine midbrain raphe. Biol. Psychiatry.

[55] Hiroi R., McDevitt R.A., Neumaier J.F. (2006). Estrogen selectively increases tryptophan hydroxylase-2 mRNA expression in distinct subregions of rat midbrain raphe nucleus: Association between gene expression and anxiety behavior in the open field. Biol. Psychiatry.

[56] Lu N.Z., Bethea C.L. (2002). Ovarian steroid regulation of 5-HT1A receptor binding and g protein activation in female monkeys. Neuropsychopharmacology.

[57] Sell S.L., Craft R.M., Seitz P.K., Stutz S.J., Cunningham K.A., Thomas M.L. (2008). Estradiol-sertraline synergy in ovariectomized rats. Psychoneuroendocrinology.

[58] Westlund Tam L., Parry B.L. (2003). Does estrogen enhance the antidepressant effects of fluoxetine?. J. Affect. Disord..

[59] Schneider L.S., Small G.W., Hamilton S.H., Bystritsky A., Nemeroff C.B., Meyers B.S. (1997). Estrogen replacement and response to fluoxetine in a multicenter geriatric depression trial. Fluoxetine collaborative study group. Am. J. Geriatr. Psychiatry.

[60] Kono S., Sunagawa Y., Higa H., Sunagawa H. (1990). Age of menopause in japanese women: Trends and recent changes. Maturitas.

[61] Mansoor B., Rengasamy M., Hilton R., Porta G., He J., Spirito A., Emslie G.J., Mayes T.L., Clarke G., Wagner K.D. (2013). The bidirectional relationship between body mass index and treatment outcome in adolescents with treatment-resistant depression. J. Child Adolesc. Psychopharmacol..

[62] Tschritter O., Preissl H., Hennige A.M., Stumvoll M., Porubska K., Frost R., Marx H., Klosel B., Lutzenberger W., Birbaumer N. (2006). The cerebrocortical response to hyperinsulinemia is reduced in overweight humans: A magnetoencephalographic study. Proc. Natl. Acad. Sci. USA.

[63] Ryan J.P., Sheu L.K., Critchley H.D., Gianaros P.J. (2012). A neural circuitry linking insulin resistance to depressed mood. Psychosom. Med..

[64] Kullmann S., Heni M., Veit R., Scheffler K., Machann J., Haring H.U., Fritsche A., Preissl H. (2015). Selective insulin resistance in homeostatic and cognitive control brain areas in overweight and obese adults. Diabetes Care.

[65] Anthony K., Reed L.J., Dunn J.T., Bingham E., Hopkins D., Marsden P.K., Amiel S.A. (2006). Attenuation of insulin-evoked responses in brain networks controlling appetite and reward in insulin resistance: The cerebral basis for impaired control of food intake in metabolic syndrome?. Diabetes.

[66] Kullmann S., Heni M., Veit R., Ketterer C., Schick F., Haring H.U., Fritsche A., Preissl H. (2012). The obese brain: Association of body mass index and insulin sensitivity with resting state network functional connectivity. Hum. Brain Mapp..

[67] Giacobbe P., Mayberg H.S., Lozano A.M. (2009). Treatment resistant depression as a failure of brain homeostatic mechanisms: Implications for deep brain stimulation. Exp. Neurol..

[68] Gasquoine P.G. (2013). Localization of function in anterior cingulate cortex: From psychosurgery to functional neuroimaging. Neurosci. Biobehav. Rev..

[69] Hauptman J.S., DeSalles A.A., Espinoza R., Sedrak M., Ishida W. (2008). Potential surgical targets for deep brain stimulation in treatment-resistant depression. Neurosurg. Focus.

[70] Lener M.S., Iosifescu D.V. (2015). In pursuit of neuroimaging biomarkers to guide treatment selection in major depressive disorder: A review of the literature. Ann. N. Y. Acad. Sci..

[71] Musselman D.L., Betan E., Larsen H., Phillips L.S. (2003). Relationship of depression to diabetes types 1 and 2: Epidemiology, biology, and treatment. Biol. Psychiatry.

[72] Drevets W.C. (2000). Neuroimaging studies of mood disorders. Biol. Psychiatry.

[73] Osby U., Brandt L., Correia N., Ekbom A., Sparen P. (2001). Excess mortality in bipolar and unipolar disorder in sweden. Arch. Gen. Psychiatry.

[74] McIntyre R.S., Soczynska J.K., Konarski J.Z., Woldeyohannes H.O., Law C.W., Miranda A., Fulgosi D., Kennedy S.H. (2007). Should depressive syndromes be reclassified as “metabolic syndrome type II”?. Ann. Clin. Psychiatry.

[75] Craft S., Watson G.S. (2004). Insulin and neurodegenerative disease: Shared and specific mechanisms. Lancet Neurol..

[76] McEwen B.S., Magarinos A.M., Reagan L.P. (2002). Studies of hormone action in the hippocampal formation: Possible relevance to depression and diabetes. J. Psychosom. Res..

[77] Masters B.A., Shemer J., Judkins J.H., Clarke D.W., le Roith D., Raizada M.K. (1987). Insulin receptors and insulin action in dissociated brain cells. Brain Res..

[78] Shibata S., Liou S.Y., Ueki S., Oomura Y. (1986). Inhibitory action of insulin on suprachiasmatic nucleus neurons in rat hypothalamic slice preparations. Physiol. Behav..

[79] Broderick P.A., Jacoby J.H. (1989). Central monoamine dysfunction in diabetes: Psychotherapeutic implications: Electroanalysis by voltammetry. Acta Physiol. Pharmacol. Latinoam..

[80] Chen C.C., Yang J.C. (1991). Effects of short and long-lasting diabetes mellitus on mouse brain monoamines. Brain Res..

[81] Lackovic Z., Salkovic M., Kuci Z., Relja M. (1990). Effect of long-lasting diabetes mellitus on rat and human brain monoamines. J. Neurochem..

[82] Sandrini M., Vitale G., Vergoni A.V., Ottani A., Bertolini A. (1997). Streptozotocin-induced diabetes provokes changes in serotonin concentration and on 5-HT1A and 5-HT2 receptors in the rat brain. Life Sci..

[83] Li J.X., France C.P. (2008). Food restriction and streptozotocin treatment decrease 5-HT1A and 5-HT2A receptor-mediated behavioral effects in rats. Behav. Pharmacol..

[84] Yanagita T., Nemoto T., Satoh S., Yoshikawa N., Maruta T., Shiraishi S., Sugita C., Murakami M. (2013). Neuronal insulin receptor signaling: A potential target for the treatment of cognitive and mood disorders. INTECH Open Access Ment. Behav. Disord. Dis. Nervous Syst..

[85] Duman R.S., Voleti B. (2012). Signaling pathways underlying the pathophysiology and treatment of depression: Novel mechanisms for rapid-acting agents. Trends Neurosci..

[86] Dandona P., Aljada A., Chaudhuri A., Mohanty P., Garg R. (2005). Metabolic syndrome: A comprehensive perspective based on interactions between obesity, diabetes, and inflammation. Circulation.

[87] Kloting N., Bluher M. (2014). Adipocyte dysfunction, inflammation and metabolic syndrome. Rev. Endocr. Metab. Disord..

[88] Xu H., Barnes G.T., Yang Q., Tan G., Yang D., Chou C.J., Sole J., Nichols A., Ross J.S., Tartaglia L.A. (2003). Chronic inflammation in fat plays a crucial role in the development of obesity-related insulin resistance. J. Clin. Investig..

[89] Quan N., Banks W.A. (2007). Brain-immune communication pathways. Brain Behav. Immun..

[90] Goehler L.E., Erisir A., Gaykema R.P. (2006). Neural-immune interface in the rat area postrema. Neuroscience.

[91] Shelton R.C., Miller A.H. (2011). Inflammation in depression: Is adiposity a cause?. Dialogues Clin. Neurosci..

[92] Shelton R.C., Miller A.H. (2010). Eating ourselves to death (and despair): The contribution of adiposity and inflammation to depression. Prog. Neurobiol..

[93] Yamada N., Katsuura G., Ochi Y., Ebihara K., Kusakabe T., Hosoda K., Nakao K. (2011). Impaired cns leptin action is implicated in depression associated with obesity. Endocrinology.

[94] Haroon E., Raison C.L., Miller A.H. (2012). Psychoneuroimmunology meets neuropsychopharmacology: Translational implications of the impact of inflammation on behavior. Neuropsychopharmacology.

[95] Kemp D.E., Ismail-Beigi F., Ganocy S.J., Conroy C., Gao K., Obral S., Fein E., Findling R.L., Calabrese J.R. (2012). Use of insulin sensitizers for the treatment of major depressive disorder: A pilot study of pioglitazone for major depression accompanied by abdominal obesity. J. Affect. Disord..

[96] Leboyer M., Soreca I., Scott J., Frye M., Henry C., Tamouza R., Kupfer D.J. (2012). Can bipolar disorder be viewed as a multi-system inflammatory disease?. J. Affect. Disord..

[97] Jun T.Y., Pae C.U., Hoon H., Chae J.H., Bahk W.M., Kim K.S., Serretti A. (2003). Possible association between -G308A tumour necrosis factor-α gene polymorphism and major depressive disorder in the korean population. Psychiatr. Genet..

[98] Yu Y.W., Chen T.J., Hong C.J., Chen H.M., Tsai S.J. (2003). Association study of the interleukin-1 β (C-511T) genetic polymorphism with major depressive disorder, associated symptomatology, and antidepressant response. Neuropsychopharmacology.

[99] Benedetti F., Lucca A., Brambilla F., Colombo C., Smeraldi E. (2002). Interleukine-6 serum levels correlate with response to antidepressant sleep deprivation and sleep phase advance. Prog. Neuropsychopharmacol. Biol. Psychiatry.

[100] Yoshimura R., Hori H., Ikenouchi-Sugita A., Umene-Nakano W., Ueda N., Nakamura J. (2009). Higher plasma interleukin-6 (IL-6) level is associated with SSRI- or SNRI-refractory depression. Prog. Neuropsychopharmacol. Biol. Psychiatry.

[101] Miller G.E., Stetler C.A., Carney R.M., Freedland K.E., Banks W.A. (2002). Clinical depression and inflammatory risk markers for coronary heart disease. Am. J. Cardiol..

[102] Thomas A.J., Davis S., Morris C., Jackson E., Harrison R., O‘Brien J.T. (2005). Increase in interleukin-1β in late-life depression. Am. J. Psychiatry.

[103] Aguirre V., Uchida T., Yenush L., Davis R., White M.F. (2000). The c-jun NH_2_-terminal kinase promotes insulin resistance during association with insulin receptor substrate-1 and phosphorylation of Ser^307^. J. Biol. Chem..

[104] Pomytkin I.A., Cline B.H., Anthony D.C., Steinbusch H.W., Lesch K.P., Strekalova T. (2015). Endotoxaemia resulting from decreased serotonin tranporter (5-HTT) function: A reciprocal risk factor for depression and insulin resistance?. Behav. Brain Res..

[105] Weichhart T., Saemann M.D. (2008). The PI3K/Akt/mTOR pathway in innate immune cells: Emerging therapeutic applications. Ann. Rheum. Dis..

[106] Kitagishi Y., Kobayashi M., Kikuta K., Matsuda S. (2012). Roles of PI3K/Akt/GSK3/mTOR pathway in cell signaling of mental illnesses. Depression Res. Treat..

[107] Kim J.Y., Duan X., Liu C.Y., Jang M.H., Guo J.U., Pow-anpongkul N., Kang E., Song H., Ming G.L. (2009). DISC1 regulates new neuron development in the adult brain via modulation of Akt-mTOR signaling through KIAA1212. Neuron.

[108] Beaulieu J.M., Gainetdinov R.R., Caron M.G. (2009). Akt/GSK3 signaling in the action of psychotropic drugs. Annu. Rev. Pharmacol. Toxicol..

[109] Sheppard K., Kinross K.M., Solomon B., Pearson R.B., Phillips W.A. (2012). Targeting PI3 kinase/Akt/mTOR signaling in cancer. Crit. Rev. Oncog..

[110] Okumura N., Yoshida H., Kitagishi Y., Murakami M., Nishimura Y., Matsuda S. (2012). PI3K/Akt/PTEN signaling as a molecular target in leukemia angiogenesis. Adv. Hematol..

[111] Fried S.K., Bunkin D.A., Greenberg A.S. (1998). Omental and subcutaneous adipose tissues of obese subjects release interleukin-6: Depot difference and regulation by glucocorticoid. J. Clin. Endocrinol. Metab..

[112] Fleet-Michaliszyn S.B., Soreca I., Otto A.D., Jakicic J.M., Fagiolini A., Kupfer D.J., Goodpaster B.H. (2008). A prospective observational study of obesity, body composition, and insulin resistance in 18 women with bipolar disorder and 17 matched control subjects. J. Clin. Psychiatry.

[113] Lee Y.H., Giraud J., Davis R.J., White M.F. (2003). C-jun N-terminal kinase (JNK) mediates feedback inhibition of the insulin signaling cascade. J. Biol. Chem..

[114] Gould T.D., Manji H.K. (2005). Glycogen synthase kinase-3: A putative molecular target for lithium mimetic drugs. Neuropsychopharmacology.

[115] Papazoglou I.K., Jean A., Gertler A., Taouis M., Vacher C.M. (2015). Hippocampal GSK3β as a molecular link between obesity and depression. Mol. Neurobiol..

[116] Girgis R.R., Javitch J.A., Lieberman J.A. (2008). Antipsychotic drug mechanisms: Links between therapeutic effects, metabolic side effects and the insulin signaling pathway. Mol. Psychiatry.

[117] Mao Z., Liu L., Zhang R., Li X. (2007). Lithium reduces FOXO3a transcriptional activity by decreasing its intracellular content. Biol. Psychiatry.

[118] Polter A., Yang S., Zmijewska A.A., van Groen T., Paik J.-H., DePinho R.A., Peng S.L., Jope R.S., Li X. (2009). Forkhead box, class o transcription factors in brain: Regulation and behavioral manifestation. Biol. Psychiatry.

[119] Huang W., Zhao Y., Zhu X., Cai Z., Wang S., Yao S., Qi Z., Xie P. (2013). Fluoxetine upregulates phosphorylated-Akt and phosphorylated-ERK1/2 proteins in neural stem cells: Evidence for a crosstalk between Akt and ERK1/2 pathways. J. Mol. Neurosci..

[120] Li X., Jope R.S. (2010). Is glycogen synthase kinase-3 a central modulator in mood regulation?. Neuropsychopharmacology.

[121] Cowen D.S., Johnson-Farley N.N., Travkina T. (2005). 5-HT1A receptors couple to activation of Akt, but not extracellular-regulated kinase (ERK), in cultured hippocampal neurons. J. Neurochem..

[122] Leone A.M., Errico M., Lin S.L., Cowen D.S. (2000). Activation of extracellular signal-regulated kinase (ERK) and Akt by human serotonin 5-HT_1B_ receptors in transfected BE(2)-C neuroblastoma cells is inhibited by RGS4. J. Neurochem..

[123] Gómez D.P.T., Velasco G., Guzman M. (2000). The CB1 cannabinoid receptor is coupled to the activation of protein kinase B/Akt. Biochem. J..

[124] Mai L., Jope R.S., Li X. (2002). BDNF-mediated signal transduction is modulated by GSK3β and mood stabilizing agents. J. Neurochem..

[125] Zheng W.-H., Kar S., Quirion R. (2002). FKHRL1 and its homologs are new targets of nerve growth factor Trk receptor signaling. J. Neurochem..

[126] Johnson-Farley N.N., Travkina T., Cowen D.S. (2006). Cumulative activation of Akt and consequent inhibition of glycogen synthase kinase-3 by brain-derived neurotrophic factor and insulin-like growth factor-1 in cultured hippocampal neurons. J. Pharmacol. Exp. Ther..

[127] Hui L., Pei D.-S., Zhang Q.-G., Guan Q.-H., Zhang G.-Y. (2005). The neuroprotection of insulin on ischemic brain injury in rat hippocampus through negative regulation of JNK signaling pathway by PI3K/Akt activation. Brain Res..

[128] Lee C.-C., Huang C.-C., Wu M.-Y., Hsu K.-S. (2005). Insulin stimulates postsynaptic density-95 protein translation via the phosphoinositide 3-kinase-Akt-mammalian target of rapamycin signaling pathway. J. Biol. Chem..

[129] Aulston B.D., Odero G.L., Glazner G.W., Aboud Z. (2013). Alzheimer’s Disease and Diabetes.

[130] Leonard B.E. (2010). The concept of depression as a dysfunction of the immune system. Curr. Immunol. Rev..

[131] Daniele S., da Pozzo E., Zappelli E., Martini C. (2015). Trazodone treatment protects neuronal-like cells from inflammatory insult by inhibiting NF-κB, p38 and JNK. Cell Signal..

[132] Encinas M., Iglesias M., Llecha N., Comella J.X. (1999). Extracellular-regulated kinases and phosphatidylinositol 3-kinase are involved in brain-derived neurotrophic factor-mediated survival and neuritogenesis of the neuroblastoma cell line SH-SY5Y. J. Neurochem..

[133] Pugazhenthi S., Nesterova A., Sable C., Heidenreich K.A., Boxer L.M., Heasley L.E., Reusch J.E. (2000). Akt/protein kinase B up-regulates Bcl-2 expression through cAMP-response element-binding protein. J. Biol. Chem..

[134] Xing J., Kornhauser J.M., Xia Z., Thiele E.A., Greenberg M.E. (1998). Nerve growth factor activates extracellular signal-regulated kinase and p38 mitogen-activated protein kinase pathways to stimulate CREB serine 133 phosphorylation. Mol. Cell. Biol..

[135] Finkbeiner S. (2000). CREB couples neurotrophin signals to survival messages. Neuron.

[136] Hardingham G.E., Fukunaga Y., Bading H. (2002). Extrasynaptic nmdars oppose synaptic nmdars by triggering CREB shut-off and cell death pathways. Nat. Neurosci..

[137] Ongur D., Drevets W.C., Price J.L. (1998). Glial reduction in the subgenual prefrontal cortex in mood disorders. Proc. Natl. Acad. Sci. USA.

[138] Anisman H., Merali Z., Hayley S. (2008). Neurotransmitter, peptide and cytokine processes in relation to depressive disorder: Comorbidity between depression and neurodegenerative disorders. Prog. Neurobiol..

[139] Perera T.D., Coplan J.D., Lisanby S.H., Lipira C.M., Arif M., Carpio C., Spitzer G., Santarelli L., Scharf B., Hen R. (2007). Antidepressant-induced neurogenesis in the hippocampus of adult nonhuman primates. J. Neurosci..

[140] Santarelli L., Saxe M., Gross C., Surget A., Battaglia F., Dulawa S., Weisstaub N., Lee J., Duman R., Arancio O. (2003). Requirement of hippocampal neurogenesis for the behavioral effects of antidepressants. Science.

[141] Myint A.M., Kim Y.K., Verkerk R., Scharpe S., Steinbusch H., Leonard B. (2007). Kynurenine pathway in major depression: Evidence of impaired neuroprotection. J. Affect. Disord..

[142] Muller N., Schwarz M.J. (2007). The immune-mediated alteration of serotonin and glutamate: Towards an integrated view of depression. Mol. Psychiatry.

[143] Moron J.A., Zakharova I., Ferrer J.V., Merrill G.A., Hope B., Lafer E.M., Lin Z.C., Wang J.B., Javitch J.A., Galli A. (2003). Mitogen-activated protein kinase regulates dopamine transporter surface expression and dopamine transport capacity. J. Neurosci..

[144] Zhu C.B., Blakely R.D., Hewlett W.A. (2006). The proinflammatory cytokines interleukin-1β and tumor necrosis factor-α activate serotonin transporters. Neuropsychopharmacology.

[145] Schwarcz R., Whetsell W.O., Mangano R.M. (1983). Quinolinic acid: An endogenous metabolite that produces axon-sparing lesions in rat brain. Science.

[146] D’Aquila P.S., Collu M., Gessa G.L., Serra G. (2000). The role of dopamine in the mechanism of action of antidepressant drugs. Eur. J. Pharmacol..

[147] Myint A.M., Kim Y.K. (2003). Cytokine-serotonin interaction through ido: A neurodegeneration hypothesis of depression. Med. Hypotheses.

[148] Chrysohoou C., Panagiotakos D.B., Pitsavos C., Skoumas I., Papademetriou L., Economou M., Stefanadis C. (2007). The implication of obesity on total antioxidant capacity in apparently healthy men and women: The attica study. Nutr. Metab. Cardiovasc. Dis..

[149] Chai W., Conroy S.M., Maskarinec G., Franke A.A., Pagano I.S., Cooney R.V. (2010). Associations between obesity and serum lipid-soluble micronutrients among premenopausal women. Nutr. Res..

[150] Galan P., Viteri F.E., Bertrais S., Czernichow S., Faure H., Arnaud J., Ruffieux D., Chenal S., Arnault N., Favier A. (2005). Serum concentrations of β-carotene, vitamins C and E, zinc and selenium are influenced by sex, age, diet, smoking status, alcohol consumption and corpulence in a general french adult population. Eur. J. Clin. Nutr..

[151] Maes M., Kubera M., Obuchowiczwa E., Goehler L., Brzeszcz J. (2011). Depression’s multiple comorbidities explained by (neuro)inflammatory and oxidative & nitrosative stress pathways. Neuro Endocrinol. Lett..

[152] Maes M., Galecki P., Chang Y.S., Berk M. (2011). A review on the oxidative and nitrosative stress (O&NS) pathways in major depression and their possible contribution to the (neuro)degenerative processes in that illness. Prog. Neuro Psychopharmacol. Biol. Psychiatry.

[153] Maes M., Vandoolaeghe E., Neels H., Demedts P., Wauters A., Meltzer H.Y., Altamura C., Desnyder R. (1997). Lower serum zinc in major depression is a sensitive marker of treatment resistance and of the immune/inflammatory response in that illness. Biol. Psychiatry.

[154] Maes M., Mihaylova I., Kubera M., Uytterhoeven M., Vrydags N., Bosmans E. (2009). Lower plasma coenzyme Q10 in depression: A marker for treatment resistance and chronic fatigue in depression and a risk factor to cardiovascular disorder in that illness. Neuro Endocrinol. Lett..

[155] Maes M., Mihaylova I., Kubera M., Leunis J.C., Geffard M. (2011). IgM-mediated autoimmune responses directed against multiple neoepitopes in depression: New pathways that underpin the inflammatory and neuroprogressive pathophysiology. J. Affect. Disord..

[156] Moylan S., Maes M., Wray N.R., Berk M. (2013). The neuroprogressive nature of major depressive disorder: Pathways to disease evolution and resistance, and therapeutic implications. Mol. Psychiatry.

[157] Arlt S., Kontush A., Muller-Thomsen T., Beisiegel U. (2001). Lipid peroxidation as a common pathomechanism in coronary heart disease and Alzheimer disease. Z. Gerontol. Geriatr..

[158] Sultana R., Perluigi M., Butterfield D.A. (2006). Protein oxidation and lipid peroxidation in brain of subjects with Alzheimer’s disease: Insights into mechanism of neurodegeneration from redox proteomics. Antioxid. Redox Signal..

[159] Greilberger J., Koidl C., Greilberger M., Lamprecht M., Schroecksnadel K., Leblhuber F., Fuchs D., Oettl K. (2008). Malondialdehyde, carbonyl proteins and albumin-disulphide as useful oxidative markers in mild cognitive impairment and Alzheimer’s disease. Free Radic. Res..

[160] Maes M., Ruckoanich P., Chang Y.S., Mahanonda N., Berk M. (2011). Multiple aberrations in shared inflammatory and oxidative & nitrosative stress (IO&NS) pathways explain the co-association of depression and cardiovascular disorder (CVD), and the increased risk for CVD and due mortality in depressed patients. Prog. Neuro Psychopharmacol. Biol. Psychiatry.

[161] Matute C., Domercq M., Sanchez-Gomez M.V. (2006). Glutamate-mediated glial injury: Mechanisms and clinical importance. Glia.

[162] Ida T., Hara M., Nakamura Y., Kozaki S., Tsunoda S., Ihara H. (2008). Cytokine-induced enhancement of calcium-dependent glutamate release from astrocytes mediated by nitric oxide. Neurosci. Lett..

[163] Bengesser S.A., Lackner N., Birner A., Fellendorf F.T., Platzer M., Mitteregger A., Unterweger R., Reininghaus B., Mangge H., Wallner-Liebmann S.J. (2015). Peripheral markers of oxidative stress and antioxidative defense in euthymia of bipolar disorder—Gender and obesity effects. J. Affect. Disord..

[164] Sarandol A., Sarandol E., Eker S.S., Erdinc S., Vatansever E., Kirli S. (2007). Major depressive disorder is accompanied with oxidative stress: Short-term antidepressant treatment does not alter oxidative-antioxidative systems. Hum. Psychopharmacol..

[165] Andreazza A.C., Frey B.N., Erdtmann B., Salvador M., Rombaldi F., Santin A., Goncalves C.A., Kapczinski F. (2007). DNA damage in bipolar disorder. Psychiatry Res..

[166] Vargas H.O., Nunes S.O., Pizzo de Castro M., Bortolasci C.C., Sabbatini Barbosa D., Kaminami Morimoto H., Venugopal K., Dodd S., Maes M., Berk M. (2013). Oxidative stress and lowered total antioxidant status are associated with a history of suicide attempts. J. Affect. Disord..

[167] McCloughen A., Foster K. (2011). Weight gain associated with taking psychotropic medication: An integrative review. Int. J. Ment. Health Nurs..

[168] Levitan R.D., Davis C., Kaplan A.S., Arenovich T., Phillips D.I., Ravindran A.V. (2012). Obesity comorbidity in unipolar major depressive disorder: Refining the core phenotype. J. Clin. Psychiatry.

[169] Vogelzangs N., Beekman A.T., Boelhouwer I.G., Bandinelli S., Milaneschi Y., Ferrucci L., Penninx B.W. (2011). Metabolic depression: A chronic depressive subtype? Findings from the inchianti study of older persons. J. Clin. Psychiatry.

[170] Yim C.Y., Soczynska J.K., Kennedy S.H., Woldeyohannes H.O., Brietzke E., McIntyre R.S. (2012). The effect of overweight/obesity on cognitive function in euthymic individuals with bipolar disorder. Eur. Psychiatry.

[171] Rosenblat J.D., Brietzke E., Mansur R.B., Maruschak N.A., Lee Y., McIntyre R.S. (2015). Inflammation as a neurobiological substrate of cognitive impairment in bipolar disorder: Evidence, pathophysiology and treatment implications. J. Affect. Disord..

